# Genetic Abnormalities in Chronic Lymphocytic Leukemia: Where We Are and Where We Go

**DOI:** 10.1155/2014/435983

**Published:** 2014-05-22

**Authors:** Anna Puiggros, Gonzalo Blanco, Blanca Espinet

**Affiliations:** ^1^Laboratori de Citogenètica Molecular, Servei de Patologia, Hospital del Mar, Passeig Marítim 25-29, 08003 Barcelona, Spain; ^2^GRETNHE, Programa de Recerca en Càncer, Institut Hospital del Mar d'Investigacions Mèdiques, Doctor Aiguader 88, 08003 Barcelona, Spain; ^3^Department of Experimental and Health Sciences, Universitat Pompeu Fabra, Doctor Aiguader 88, 08003 Barcelona, Spain

## Abstract

Chromosomal abnormalities in chronic lymphocytic leukemia (CLL) are detected in up to 80% of patients. Among them, deletions of 11q, 13q, 17p, and trisomy 12 have a known prognostic value and play an important role in CLL pathogenesis and evolution, determining patients outcome and therapeutic strategies. Standard methods used to identify these genomic aberrations include both conventional G-banding cytogenetics (CGC) and fluorescence *in situ* hybridization (FISH). Although FISH analyses have been implemented as the gold standard, CGC allows the identification of chromosomal translocations and complex karyotypes, the latest associated with poor outcome. Genomic arrays have a higher resolution that allows the detection of cryptic abnormalities, although these have not been fully implemented in routine laboratories. In the last years, next generation sequencing (NGS) methods have identified a wide range of gene mutations (e.g., *TP53, NOTCH1, SF3B1,* and *BIRC3*) which have improved our knowledge about CLL development, allowing us to refine both the prognostic subgroups and better therapeutic strategies. Clonal evolution has also recently arisen as a key point in CLL, integrating cytogenetic alterations and mutations in a dynamic model that improve our understanding about its clinical course and relapse.

## 1. Introduction

Chronic lymphocytic leukemia (CLL) is the most common leukemia of adults in Western countries. The clinical course is highly variable, ranging from very indolent cases to patients with aggressive and rapidly progressing disease. This heterogeneity has important consequences which will impact on clinical approaches, treatment strategies, and, finally, survival times from diagnosis [1].

Acquired genetic aberrations, as in other types of cancer, have an important role in CLL pathogenesis. Since the late 1970s, numerous genetic studies using a wide range of laboratory techniques (conventional G-banding cytogenetics, fluorescence* in situ* hybridization, microsatellite analysis to detect loss of heterozygosity, Sanger sequencing, genomic arrays, and more recently next generation sequencing methodologies, among others) have identified a broad spectrum of genomic aberrations. Overall, taking into account all of these data, CLL is characterized by a relatively stable genome, in comparison with other hematological malignancies or solid tumors. At diagnosis, 80% of cases show between 0 and 2 copy number alterations and the remaining 20% harbor ≥3 [2, 3]. The most frequent chromosomal abnormalities are partial losses of one affected chromosome, such as deletions on 6q, 11q, 13q, or 17p; gains of entire chromosomes, such as trisomy 12, are less frequent. In addition, balanced translocations can also be detected in a proportion of cases [4]. Since the late 1990s, there is an evidence that certain cytogenetic abnormalities, such as 11q or 17p deletions, are associated with a poor clinical outcome and have become important prognostic factors [2]. However, chromosomal aberrations may not be responsible for the whole clinical heterogeneity of CLL. Mutations in certain genes, as* TP53*, are accountable for poor prognosis. Recent studies using next generation sequencing techniques have allowed the identification of new genomic abnormalities, such as* NOTCH1* and* SF3B1* mutations as well as* BIRC3* disruptions, which may explain part of the clinical heterogeneity and open the door to new therapeutic strategies.

The aim of the present review is to summarize the main genetic abnormalities identified in CLL patients, to describe their impact in daily clinical practice, and to discuss the recent findings using novel techniques.

## 2. Cytogenetic Aberrations with Known Prognostic Value

### 2.1. 13q14 Deletion

Deletion of 13q14 region, found in more than 50% of CLL patients, is the most common cytogenetic abnormality detected by fluorescence* in situ* hybridization (FISH) and has historically been associated with good prognosis. During the last years, several studies have revealed some insights in the candidate genes located at 13q that could be responsible for CLL pathogenesis, as well as in the prognostic heterogeneity of 13q-deleted patients.

**Figure 1:**
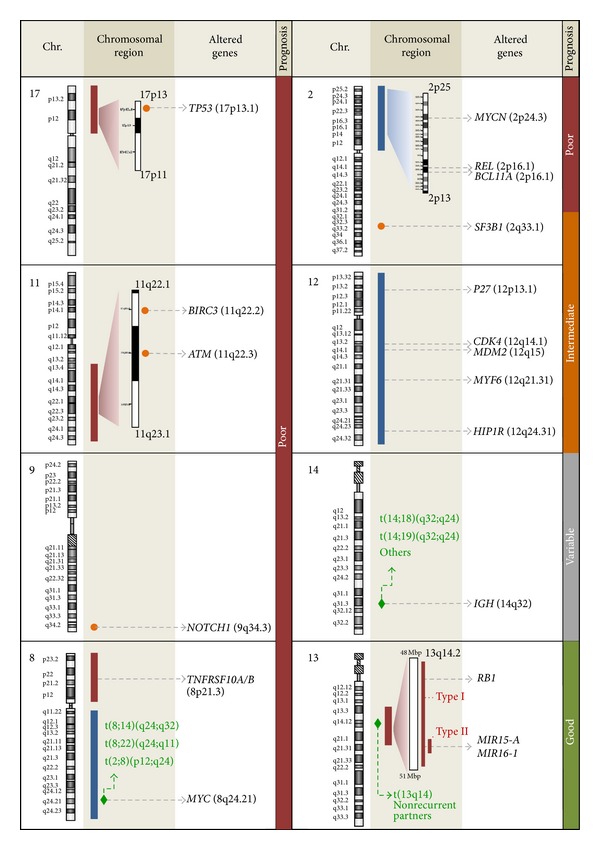
Main genetic abnormalities with known prognostic significance in CLL. Genetic abnormalities are grouped by chromosomes (Chr.). In the chromosomal region section, losses and gains are represented in red and blue bars, respectively; breakpoints for translocations are depicted as green diamonds; loci where recurrently mutated genes are located are shown in orange circles.

With regard to biologic basis underlying 13q deletions, miR-15a and miR16-1, located in the minimal deleted region (MDR), have been described to exhibit a tumoral suppressor function in CLL [5, 6]. However, miR-15a and miR16-1 are not invariably included in 13q deletions, and although their expression is decreased in several CLL patients, a clear correlation with the number of deleted 13q alleles has not been found [7]. Thereby, besides these microRNAs, other genes located in 13q, such as* DLEU7*, could cooperate in the tumoral suppressor activity. In addition, it has been extensively demonstrated that large 13q losses involving* RB1* (type I deletions) gene are related to shorter time to first treatment (TTFT) and overall survival (OS) than those small deletions encompassing only miR-15a and miR16-1 (type II) (Figure 1 and Table 1) [7–10].

In contrast to other recurrent abnormalities in CLL, the presence of biallelic losses in 13q has been described in nearly 30% of 13q-deleted CLL patients [16]. Biallelic 13q deletions are characteristically small and do not involve* RB1* [7]; nevertheless their clinical impact has been controversial. Some authors hypothesized that they result from an evolution of the monoallelic losses and therefore represent a more aggressive abnormality [13, 14, 17–19]. However, we and others did not find significant differences in the baseline characteristics and clinical outcome among CLL patients with monoallelic or biallelic 13q deletions [13, 20, 21]. It is worth noting that 13q14 region can be inactivated by other mechanisms such as copy neutral loss of heterozygosity [3] and epigenetic silencing by DNA methylation of CpG islands [22] or histone deacetylation [23]. Thus, it is feasible to assume that the potential effect of the biallelic 13q losses on the prognosis could be masked either by the size of the deleted region or the inactivation of the remaining allele by other mechanisms.

Regarding the size of the abnormal clone detected by FISH, it has been described that those patients with a higher percentage of altered nuclei have a significantly shorter TTFT and OS. The optimal cut-off point that defines a poorer outcome of 13q deletion differs between published studies [12–14, 19, 21]. Indeed, although the cut-off described ranged from 65.5% to 90%, the percentage of 13q deletion had a predictive value as a continuous variable [13, 19]. Thus, those patients with isolated 13q deletion can be risk-stratified according to the percentage of altered cells by FISH (Table 1).

Biologic heterogeneity underlying clinical differences observed among 13q-deleted patients has been also demonstrated by gene expression profiling and miRNA analyses [8, 24]. Specific transcriptional profiles have been correlated with two subgroups of 13q deletion based on the size of deleted area (short/biallelic versus wide/monoallelic). Thus, those patients with large 13q losses showed downregulation of ten genes including* TPT1/TCTP*, which is involved in prosurvival and growth signaling through inhibition of BAX-induced apoptosis and overexpression of 53 genes. Most of the upregulated genes (*AMF, GPI, BSG, LGALS1, PAK2, PARVB, *and* VIM*, among others) were involved in cell motility and adhesion, regulation of cell proliferation, tumor cell migration, metastasis, angiogenesis, and apoptosis. Interestingly, deregulation of many relevant cellular pathways has also been shown in those patients with higher percentages of 13q deletion (above 80%). Among them are remarkably the deregulation of several important miRNAs and overexpression of genes mainly involved in BCR signaling (e.g.,* SYK *and* CD79b)*, NF*κ*B signaling, and prosurvival and antiapoptotic pathways (e.g., Wnt and RAS signaling). Of note, the gene expression pattern observed in patients with >80% of 13q-deleted cells was similar to the patients with 11q- and 17p- included in the study [24].

In all, although heterogeneity of 13q deletion has been demonstrated, recent studies that integrate molecular and cytogenetic data keep on considering patients with isolated 13q deletion as a very low-risk group [25, 26]. Moreover, Jeromin et al. found association with mutations in* MYD88*, which have no apparent prognostic effect [25].

### 2.2. Trisomy 12

Trisomy 12 is the third most frequent chromosomal aberration in CLL (10–20% of cases) and often appears as the unique cytogenetic alteration (40–60% of cases with +12). Besides, it can be associated with other chromosomal aberrations, including trisomies 18 and 19, recurrent CLL deletions (e.g., 14q, 13q, 11q, or 17p), and* IGH* translocations [2, 27].

Trisomy 12 was considered as an intermediate risk marker in the hierarchical prognostic model initially proposed by Döhner et al. [2]. However, this category still remains quite controversial. Whereas first studies often correlated trisomy 12 with a more aggressive clinical course [28, 29], recent publications tend to include it in an intermediate or even low-risk category [26, 30]. Regardless, it corresponds with a clinical heterogeneous entity. Recently,* NOTCH1 *has emerged as a strongly associated marker that presents a high mutation frequency in +12 CLL patients, especially in those having a bad outcome (with unmutated* IGHV *genes and/or ZAP70+). The higher frequency of mutated* NOTCH1*, as well as CD38 or CD49d expression, could explain at least in part the bad prognosis of these subgroups of cases and thus the different survival rates in the entire +12 cohort (Figure 1 and Table 1) [31–33]. Trisomy 12 is mainly considered as a clonal driver mutation that occurs early in CLL evolution and facilitates the appearance of secondary chromosomal aberrations or mutations in genes as* NOTCH1*,* TP53, *and* FBXW7* [34, 35]. Thus, it has been shown that cases with +12 and mutated* IGHV* genes could easily acquire an additional chromosome 19 [36]. Recently, this subgroup of patients has been associated with very high CD38 expression (even more than +12 isolated cases), which occurs exclusively in isotype-switched IgG cells, a rare immunoglobulin variant in CLL [37]. Atypical morphology and immunophenotype have also been related to trisomy 12 [31, 38]. Moreover, higher frequencies of unmutated* IGHV* genes, as well as* IGHV1 *and* IGHV4-39* variants, have been described in patients carrying +12 [35, 38, 39].

Regarding the pathogenesis of trisomy 12, it has been difficult to establish a set of candidate genes since the affected region is the whole chromosome instead of a smaller critical region. Nonetheless, RNA and protein expression analysis have suggested a gene dosage effect [40–42]. As expected, trisomy 12 is associated with an upregulation of genes distributed along the whole chromosome, such as* P27, CDK4*,* HIP1R*,* MYF6,* and* MDM2* [42, 43].* HIP1R *overexpression has been proposed as the best potential surrogate marker, although its clinical relevance remains uncertain [43]. Besides,* MDM2 *is involved inp53 degradation; thus its overexpression can lead to cell cycle deregulation in patients harboring this alteration [44]. In addition, other genes not located on chromosome 12 have been described to be differentially expressed in this subgroup of patients, mainly overexpressed, such as* BAX* or* E2F1. E2F1* is a transcription factor associated with proliferation and its activity is regulated through kinases such as* CDK4, *located on 12q14.1. Therefore, a direct gene dosage-dependent upregulation of* CDK4* might contribute to* E2F1* overexpression in trisomy 12 patients, which suggests an increased proliferative activity as a potential pathomechanism in the course of this chromosomal aberration [42]. Furthermore,* CD200* and* P2RY14* are underexpressed in trisomy 12 cases, which suggests that additional transacting interactions might play an important role in the evolution of this group of CLL patients [43]. However, a detailed pathomechanism of trisomy 12 has not been fully elucidated (Figure 1).

### 2.3. 11q23 Deletion

Deletion of the long arm of chromosome 11 is detected in 5–20% of CLL patients [2, 15, 45]. These deletions are highly variable in size, being larger than 20 megabases in most cases [46, 47]. The MDR includes 11q22.3-q23.1 chromosome bands, thus harboring the* ATM* gene in almost all cases, as well as other genes including* RDX, FRDX1, RAB39, CUL5, ACAT, NPAT, KDELC2, EXPH2, MRE11, H2AX,* and* BIRC3 *(Figure 1). Indeed, cases can be classified in “classical or large deletion” (more common) and “atypical or small deletion” (uncommon and more frequently associated with* ATM* mutations). It is remarkable that no homozygous 11q deletions have been described. Regarding the association between del(11q) and other chromosomal abnormalities, such cases show an increased copy number alterations, thus indicating genomic instability [15, 48].* ATM* gene mutations have been largely studied in CLL patients with del(11q); however, they have been found in only 8–30% of 11q- patients [49], indicating that other genes could play a role in the pathobiology of 11q deletions in CLL. One of these genes is* BIRC3*, which is located near to* ATM* gene, at 11q22.* BIRC3* disrupting mutations and deletions have been rarely detected in CLL at diagnosis (4%) but detected in 24% of fludarabine-refractory CLL patients (Figure 1). Interestingly, progressive but fludarabine-sensitive patients did not show* BIRC3* aberrations, suggesting that* BIRC3* genetic lesions are specifically associated with a chemorefractory CLL phenotype [50]. In addition, it is remarkable that patients with* BIRC3* lesions are mutually exclusive with CLL patients harboring* TP53* abnormalities. However, a recent study by Rose-Zerilli et al. has shown that* ATM* mutations rather than* BIRC3 *deletion and/or mutation had impact on overall and progression-free survival in 11q-deleted CLL patients treated with first-line therapy [51].

From a clinical point of view, CLL patients with del(11q) are characterized by large and multiple lymphadenopathies and have been associated with poor prognostic factors, such as unmutated* IGHV* genes. Regarding the prognostic significance, the presence of del(11q) implies clinically progressive disease in almost all cases. In addition, those 11q- cases have been associated with shorter TTFT, shorter remission durations, and shorter OS following standard chemotherapy compared to nondeleted 11q (and nondeleted 17p) cases [52]. However, more recent treatments based on chemoimmunotherapy may overcome the adverse prognostic significance of 11q deletion in previously untreated patients. It has been reported that del(11q) does not have impact on progression-free survival (PFS); however, there is still a lack of information regarding long-term OS studies. Moreover, genetic heterogeneity displayed in patients with del(11q) may impact on long-term clinical outcome (Table 1) [53].

### 2.4. 17p13 Deletion

Deletion of 17p is found in approximately 3–8% of CLL patients at diagnosis [2, 54, 55]. However, it can account for up to 30% in patients treated with chemotherapy and undergo refractory CLL [55, 56]. Thus, it is one of the most frequently acquired aberrations triggered after treatment, not only in CLL but also in other non-Hodgkin's lymphomas such us mantle cell lymphoma or diffuse large B-cell lymphoma.

Patients with 17p deletion have always been included into the highest risk prognostic category, showing the shortest OS and PFS. This finding can be explained not only because of the cell-cycle deregulation caused by the loss of* TP53 *but also the usual requirement of chemotherapy, both independent predictors of a reduced OS and PFS (Table 1) [26, 57]. Nonetheless, recent studies have shown clinical heterogeneity in 17p- patients according to the appearance of this abnormality during follow-up: as an early event (*de novo*) or, more frequent, as a secondary alteration [34]. Patients with* de novo* 17p- have a longer median OS (4-5 years) whereas those who acquired 17p- during clonal evolution have a notably decreased survival (1–1.5 years) [11]. Another important factor that stratifies 17p deletion patients in different risk subgroups is the percentage of nuclei altered by FISH. The cut-off value for the percentage of 17p-deleted nuclei that predicted poorer outcome was initially established at 3% [58], while subsequent studies increased the cut-off levels up to 25% [11, 54, 59, 60]. However, it has recently been demonstrated that the clone size has a negative impact on OS and response to treatment not only at different cut-off levels but also as a continuous variable [54]. Strikingly, latest investigations based on ultradeep next generation sequencing were able to detect* TP53* mutations in small CLL subclones that were missed by Sanger sequencing due to their very low frequency. Patients harboring small* TP53-*mutated subclones also showed a poor survival. These minority subclones became the predominant population over time and prognosticated the development of chemorefractoriness, showing the importance of* TP53* as a driver mutation [61].

It has been shown that mutations in* TP53* are frequently detected in the remaining allele of 17p- CLL patients, appearing in more than 75% of cases. This is in contrast with the low frequency of* TP53* mutations in patients without 17p deletion and may reflect a selective pressure for cells carrying biallelic inactivation of* TP53* [62]. Patients harboring both abnormalities have a significantly poorer outcome, with shorter OS and PFS and lower response rates than those with* TP53 *mutation or deletion of a single 17p allele [63, 64]. Nonetheless, del(17p) in the absence of mutated* TP53* and* vice versa* has also a negative effect on prognosis, which points that monoallelic inactivation of* TP53 *may be enough for resistance to treatment and clonal selection [63, 64]. Another mechanism that can trigger the dysfunction of* TP53* is the overexpression of* MDM2, *a p53-specific ubiquitin ligase that mediates the degradation of p53 and has a higher expression in 50% to 70% of patients [44].* MDM2* overexpression involves the repression of a large number of p53-dependent genes and miRNAs, including miR-34a, a downstream effector of p53. Although miR-34a expression is highly variable in nonaltered* TP53* cases, CLL cells from patients harboring* TP53* mutations, deletions, or* MDM2* overexpression are associated with lower levels of miR-34a. As this microRNA is involved in the regulation of senescence, apoptosis and cell cycle arrest, a more aggressive course of the disease, can be correlated with miR-34a underexpression [65]. In summary, alterations not only in* TP53* but along the whole p53 axis lead to decreased overall survival and therapy resistance in CLL.

CLL patients with 17p- have also been associated with atypical immunophenotype, with a higher intensity of CD20, FMC7, CD79b, and surface Ig [31]. In addition, an increased expression of CD38, ZAP-70, and unmutated* IGHV *was reported in 17p- cases, which agrees with the poor prognosis of this group of patients [31, 66, 67]. Other studies have demonstrated a significant correlation between 17p- and 4p-, 18p-, 20p-, or chromosome 8 alterations (8p- or 8q+) [30, 68]. Thus,* TP53* mutation/17p- is correlated with a higher genetic complexity. Nevertheless, the acquisition of these aberrations during the progression of the illness and their precise effect has not been fully elucidated.

The extent of the 17p13 deletion often encompasses most of the chromosome 17 short arm and is invariably associated with loss of* TP53* as confirmed by FISH (Figure 1). The 17p- subgroup displays the highest number of differentially expressed genes affecting apoptosis, cell cycle regulation, and BCR signaling. Underexpression of* TP53, CCND3, BCL2, SYK, ATM, TCL, PI3K, CCND1,* and* AID* and overexpression of* P2, MYC,* and* AICL* were reported in this group of patients, indicating a remarkable genetic instability. As expected,* TP53* is the most significantly downregulated gene, which underlies the molecular mechanism that confers a poor response to alkylating agents and purine analogs, although the concomitant loss of other tumor suppressor genes could be responsible for the highly adverse prognostic relevance of this subgroup [42, 69, 70].

The standard treatment for CLL is based on fludarabine-cyclophosphamide (FC) or fludarabine-cyclophosphamide-rituximab (FCR) regimens. However, patients harboring 17p deletion and/or* TP53* mutations do not respond to these therapies. In order to improve survival of nonresponding patients, a wide spectrum of new drugs acting independently of p53, either as sole agents or combined, have been tested, including flavopiridol, lenalidomide, alemtuzumab, alemtuzumab/corticosteroids, and rituximab/corticosteroids [71]. Recently, inhibitors of key pathways in tumor B-cell physiopathology have arisen as promising new drugs. Among them, dinaciclib (a cyclin-dependent kinase (CDK) inhibitor), ONO-4059 (a Bruton's tyrosine-kinase (BTK) inhibitor), ABT-199 (a Bcl-2 inhibitor), and overall ibrutinib (another BTK inhibitor) have demonstrated a significant activity in CLL [72–75]. Up to now, allogeneic stem cell transplantation was the suggested strategy for patients with 17p- who had achieved a complete remission. Nonetheless, with the confirmed activity of ibrutinib, this strategy should be reconsidered (Table 1) [71].

## 3. Other Abnormalities Described by Conventional G-Banding Cytogenetics 

Initial cytogenetic studies in CLL were highly limited by the low mitotic rate of tumoral cells in culture [76]. Indeed, interphase FISH analyses have been implemented as the gold standard for cytogenetic risk stratification of CLL patients [2]. Nonetheless, detection of abnormalities by FISH is limited to the probes used and underestimates the true complexity and heterogeneity of chromosomal aberrations in CLL. The use of new B-cell mitogens, such as CD40-ligand or CpG oligonucleotide and interleukin-2, improves the growth of CLL cells in culture, increasing the detection rate of chromosomal abnormalities up to 80% of patients [77–80]. By this method, it was demonstrated that 25–37% of patients showing no aberrations by FISH carried chromosomal abnormalities not covered by the standard FISH panel (which detects trisomy 12 and deletions of 11q, 13q, and 17p) [78, 81]. These additional abnormalities, otherwise not identified by FISH, do not correlate with other poor prognostic factors including CD38 or ZAP70 positivity and* IGHV *mutational status. However, these abnormal karyotypes were strongly correlated to advanced CLL stages and treatment requirement, as well as to worse prognosis in terms of shorter TTFT and OS [81]. Despite the high heterogeneity of the abnormalities detected, several studies have demonstrated that the number of abnormalities found by CGC can be correlated with the clinical outcome of CLL patients [4, 82–85].

### 3.1. Chromosomal Translocations

In contrast to other mature B-cell neoplasms, CLL is not characterized by the presence of specific chromosomal translocations. However, it has been described that 32–42% of CLL patients carry a wide range of translocations when studied by CGC [4, 82, 86]. The prognostic significance of these chromosomal rearrangements has been controversial. Although it was initially described that translocations were associated with poor outcome independently of the number of rearrangements [86], more recent studies restricted the poor prognosis to those cases harboring translocations in the context of a complex karyotype or unbalanced translocations [4, 82]. Unbalanced translocations preserved its prognostic significance even when analyzed in the 17p- group of patients [82]. Balanced translocations involving* IGH* are uncommon in CLL (4–9% of patients) [87]; nonetheless some* IGH* translocations have been extensively characterized in the literature. Although it was initially defined that CLL patients with* IGH* translocations were associated with poorer outcome and should be considered as a distinct prognostic group [87], subsequent studies revealed that the chromosome partner involved in the translocation could be relevant for the outcome. Thus, it has been described that t(14; 19), which involves* BCL3* locus, is associated with the presence of trisomy 12 and complex cytogenetics, unmutated* IGHV*, atypical CLL morphology, and phenotype and inferior prognosis. On the contrary, CLL patients with* IGH/BCL2* have not shown association with complex karyotype or aggressive features that could trigger a poorer outcome of this subgroup of* IGH*-translocated patients (Figure 1) [88]. Translocations involving* MYC* with* IG* or non-*IG* partners are present in less than 1% of CLL but identify a subgroup of CLL patients with higher incidence of poor prognostic features compared with general CLL population (Figure 1) [89, 90]. Moreover, as prolymphocytes are detected in most of these cases, it has been postulated that* MYC* translocations could be a secondary event with a transforming role in CLL [89, 90]. Chromosome 13q is also recurrently translocated in CLL; indeed 10% of the del(13q) identified by FISH are associated with 13q14 translocations detectable by CGC [91]. It is accepted that 13q14 has multiple chromosome partners and that the consequence of these rearrangements is the loss of a tumor suppressor gene in 13q14 [47]. Indeed, deletion of D13S319 locus has been extensively evidenced in nearly all the cases described in the literature (Figure 1) [47, 91, 92]. The impact that 13q translocations could have on the prognosis of 13q deletion is controversial. While some authors suggested that it could represent a more aggressive disease with higher incidence of* RB1* deletion [91, 92], our group has found* RB1* deletion rates similar to large cohorts of unselected CLL patients with 13q deletion (20–25%) (personal observation). Translocations in 13q are apparently balanced by CGC, and several studies have proven that balanced rearrangements do not imply a worse prognosis in CLL [4, 82, 86]. Although it was described in a limited number of patients, some authors suggested that poor prognosis of* TP53* deletions could be modified when 17p loss was caused by recurrent 17p translocations. Thus, dic(8; 17)(p11; p11) was described in four CLL patients but still have an unclear clinical impact [93], while dic(17; 18)(p11.2; p11.2) was detected in 1.3% of 1213 patients studied and was associated with early age at diagnosis and accelerated disease progression [94]. Altogether, chromosomal translocations* per se *do not confer a bad outcome in CLL. However, some recurrent rearrangements involving genes such as* BCL3*,* MYC, *or the 17p arm should be considered as poor prognostic indicators in the genetic risk stratification of patients.

### 3.2. Complex Karyotypes

Complex karyotypes (CK), defined as the presence of three or more chromosomal abnormalities, are detected in nearly 16% of patients [4, 78] and have been associated with unmutated* IGHV* status and CD38 expression [78]. Regarding prognostic significance, CK predicted shortened TTFT and OS in CLL patients treated with savage therapies, including 2-chloro-2′-deoxyadenosine (CdA) [82, 84]. Shorter OS was observed in patients with relapsed and refractory CLL treated with flavopiridol (a CDK inhibitor) [85]. As a continuous variable, the number of karyotypic abnormalities also predicted shorter event free survival (EFS) and OS in CLL patients who underwent allogeneic hematopoietic stem cell transplantation (HSCT) following reduced-intensity conditioning [83], being five abnormalities the cut-off with the highest predictive value. Of note, a highly significant association between complex aberrant karyotypes and 11q or 17p deletions has been described [78, 83]. Although it could be assumed that the prognostic significance of CK is caused by the association with these aberrations, Jaglowski et al. proved that karyotypic complexity retained its predictive value in EFS and OS even when only patients with high-risk FISH abnormalities were considered [83]. Moreover, Ouillette et al. demonstrated that genomic complexity in CLL was a consequence of an impaired DNA double-strand break response due to multiple gene defects including not only* TP53*, but also* ATM* and other genes located in 11q or* RB1* gene located at 13q14 [95]. The impact of CGC results in the outcome of CLL patients, as well as the multigenic origin of the genomic complexity in CLL; suggest that CGC should be implemented in the clinical practice to identify a subset of patients with clinical and prognostic characteristics that should be considered for the design of risk-adapted treatment strategies.

## 4. Cytogenetic Alterations Detected by Genomic Arrays

Despite being one of the first techniques used for screening of chromosomal alterations in CLL, CGC studies are limited by the requirement of dividing cells in culture and FISH analyses only offer a vision of the genome limited to the specific probes used. Microarray-based technologies have shown the ability to allow a high-resolution genome-wide exploration for allelic copy number gains and losses in CLL. Initial studies compared arrays and FISH analyses in the identification of the known recurrent CLL alterations. Overall, concordances ranging from 79% to 98% were described [48, 96–99]. The majority of discordant results were due to the lower sensitivity of genomic arrays; thus a cut-off point for the sensitivity of 20–30% of abnormal cells was fixed for different array platforms [48, 99–101]. However, subsequent studies pointed out that some deletions smaller than the probes used in the FISH analyses could be only detected by arrays [102] and algorithms based on the percentage of tumoral cells in peripheral blood have been suggested to optimize the detection of genomic abnormalities [96, 103].

On the other hand, genomic array studies have allowed the characterization of the size and the minimal deleted region of known CLL abnormalities otherwise not possible by studying CLL FISH panel. Hence, poorer outcome was described for those 13q losses comprising not only* DLEU2/MIR15A/MIR16-1* genes, but also* RB1* in the deleted area (Figure 1) [7, 10]. Gunn et al. described on their array-CGH analyses the multigenic nature of 11q deletions and the existence of a minority of 11q losses that did not involve* ATM* and could be missed by standard FISH probes [46]. Deletions of 6q, previously described in 3–6% of CLL patients by CGC and FISH [104, 105], have also been identified in several genomic array studies with a detection rate ranging from 3 to 17% [97, 102, 106]. In contrast to other known abnormalities, genomic array studies have pointed out the high heterogeneity of del(6q) and the impossibility to define an MDR for all patients. Edelmann et al. defined a region of 2.5 Mb at 6q21 that was affected in 80% of 6q- patients; however, no specific gene has been identified as responsible for the 6q- pathogenesis [102].

In addition, recurrent CLL abnormalities not included in the FISH panel, which were cryptic or identified in very low frequency by CGC, have been widely described in the genomic array studies. Schwaenen et al. initially described 2p gains including* MYCN* gene in a low proportion of CLL cases. These cases showed a significant increase of* MYCN* transcript suggesting a role in the pathogenesis of the disease [100]. Subsequent studies confirmed this overexpression and also highlighted the involvement of* REL* and* MSH2* genes in many of the 2p gains. However, none of these genes showed differential expression levels in the affected patients [106]. Regarding prognostic impact, detection of 2p gains increased up to 28% of patients in untreated Binet B/C patients and it has been postulated that 2p gains are a secondary event associated with a shorter OS and an increased risk of transformation to Richter syndrome (Figure 1) [106, 107]. An even more aggressive course was defined for those patients with association between 2p gains and the poor-prognosis 11q deletions, which triggered combination of* MYCN* overexpression and* ATM* downregulation [98]. Abnormalities in chromosome 8 (8p losses and 8q gains) described by genomic arrays have been also pointed out as prognostic markers in CLL. Although they were identified in only 2–5% of general CLL population, an increased frequency of 8p and 8q abnormalities was observed in 17p- patients, up to 80 and 44%, respectively. An independent association with shorter OS was described for these abnormalities, even when only 17p- patients were considered (Figure 1) [107, 108]. Other small recurrent abnormalities with unclear prognostic significance have been identified in genomic arrays studies; thus submicroscopic deletions in 22q11 and gains of 20q13.12 were described in 15% and 19% of CLL patients, respectively. Both abnormalities showed related gene expression changes, revealing the high diversity of genomic aberrations in CLL and identifying new candidate genes involved in the pathogenesis of the disease [109, 110].

Apart from recurrently altered regions, several authors have associated the complexity detected by genomic arrays with either shorter TTFT, worse response to therapy, or shorter OS [30, 48, 97, 101, 103]. More recently, the phenomenon of chromothripsis, in which hundreds of genomic rearrangements involving localized genomic regions occur in a single cellular crisis, has been defined in at least 2-3% of all cancers [111]. Edelmann et al. studied 353 CLL patients by genomic arrays identifying chromotripsis, defined as the presence of at least ten switches between two or three copy number states on an individual chromosome, in seven of them. Notably, patients with chromothripsis had inferior PFS and OS as well as high frequencies of unmutated* IGHV* and high-risk genomic aberrations [102].

Overall, results reported in the literature have demonstrated that microarray platforms are a valuable tool for genomic study of CLL and the identification of novel aberrations which may be cryptic by conventional techniques. Aberrations identified by arrays could be useful for a more accurate risk stratification of CLL patients and shed light on the knowledge of CLL pathogenesis. However, its incorporation in the diagnostic routine has been limited by several reasons including the lower sensitivity in the detection of known poor prognostic abnormalities, the still unknown significance of most of the small aberrations identified by genomic arrays, or the inability to detect balanced aberrations, which could be essential for the differential diagnosis with other mature B-cell neoplasms [99].

## 5. Clonal Evolution

Clonal evolution is a key point of CLL development and relapse. A recent whole-genome sequencing study identified two types of driver mutations in CLL, ones which appear as early events and were found as predominantly clonal (e.g., heterozygous 13q deletion, trisomy 12 or* MYD88,* and* NOTCH1* mutations), whereas others appear later in the course of the disease, as secondary events, and were found mainly subclonal (e.g.,* TP53, ATM*,* SF3B1* mutations, and homozygous 13q deletion) [34]. Deletions in 17p and 11q have been described both as late or early events, which could explain the heterogeneity of these subgroups of patients [11, 112]. It was also demonstrated that chemotherapy triggers clonal evolution by favoring the appearance and domination of subclones with driver mutations (such as* TP53* or* SF3B1*) which proliferate and replace the other subclones over time [34]. Nonetheless, it may take months to years for a new subclone to fully substitute those previous established [113]. Clonal devolution, defined as the disappearance of one or more clonal aberrations at follow-up, can also be observed in treated patients [55, 114, 115].

Both FISH and CGC are useful and complementary methods to study clonal evolution in CLL samples. However, FISH analyses were found to be more precise than CGC in the detection of small cytogenetic abnormalities, specially del(13q) and del(17p) [55]. A wide range of frequencies of clonal evolution has been described (10 to 45%), being the most frequently acquired abnormalities detected by these techniques high-risk aberrations (17p and 11q deletions) and mono- and biallelic deletions of 13q [55, 116]. Recently, whole-genome sequencing methods which can detect thousands of somatic mutations per subclone revealed novel early and late CLL driver mutations [34, 113]. Nevertheless, the impossibility of applying this methodology in large cohorts of patients still leaves much to be elucidated. As for the clinical relevance, it still remains quite controversial if clonal evolution has a clear impact on survival. A prognostic value for the acquisition of new genetic aberrations by itself has not been described; however, a bad prognosis was observed when high-risk lesions were acquired [55].

## 6. New Genomic Technologies and Future Perspectives

As scientific knowledge about genetics and CLL progresses, new powerful technologies have arisen, paving the way for promising findings. In the last years, next generation sequencing studies have improved our understanding about the abnormal physiopathology of tumoral B-cells as well as contributed to redefine the traditional prognostic subgroups. In addition to the previously well-characterized* TP53 *mutation, novel somatic lesions with clinical significance have been detected, most of which have already been mentioned in the present review. Among them, alterations in* NOTCH1*,* SF3B1*,* BIRC3*,* ATM,* and* MYD88 *were recurrently found, appearing in 3–15% of CLL patients, either combined or as solely lesions [25, 26, 117, 118]. The appearance of these mutations is a consequence of the dynamic dialogue between CLL cells and microenvironment selective pressures, which widely determines clonal evolution and response to treatment [34].

Prognosis prediction models usually employed to stratify CLL patients include clinical factors (mainly based on the lymphocyte doubling time and Rai and Binet staging systems), molecular markers (expression of CD38, ZAP-70, and* IGHV* mutational status), and chromosomal abnormalities. With the new findings provided by NGS, the cytogenetic model was proposed to be refined by integrating the analysis of recurrent gene mutations, since most of them demonstrated to have a clinical independent impact on patient survival. In a recent study published by Rossi et al., a four-category model was proposed: high risk (patients harboring del(17p)/*TP53* mutation and/or* BIRC3* mutation), intermediate risk (harboring del(11q),* NOTCH1* mutation, and/or* SF3B1* mutation), low risk (harboring trisomy 12 or normal karyotype), and very low risk (if del(13q) is present as the sole abnormality) (Table 1). In addition, the proportion of the abnormal clone is gaining importance in the stratification of already defined groups, as several studies have reported different outcomes in patients with high or low percentages of nuclei harboring 13q-, 11q-, and 17p- by FISH [11, 12, 15]. In conclusion, the current prognostic models based on genetic abnormalities are nowadays subject to change as new cytogenetic and mutational findings are revealed, contributing to refine better and better these approaches.

## Figures and Tables

**Table 1:** Prognostic subgroups and associated risk genetic factors in CLL at diagnosis.

Category	Associated genetic factors	Therapeutic strategies
Very high risk	del(17p)*/*TP53* mutation and/or *BIRC3* mutation	p53-independent drugs, BTK inhibitors, allogeneic stem cell transplantation

High risk	del(11q)*/*ATM* mutation and/or *NOTCH1* mutation and/or *SF3B1* mutation	FCR

Intermediate risk	Trisomy 12 Normal karyotype and FISH	Not recommended

Low risk	Isolated del(13q)*	Not recommended

FCR: fludarabine, cyclophosphamide, and rituximab; *higher percentages of deleted nuclei have bad impact on prognosis (Tam et al., 2009 [11]; Hernández et al., 2009 [12]; van Dyke et al., 2010 [13]; Dal Bo et al., 2011 [14]; Marasca et al., 2013 [15]; Puiggros et al., 2013 [21]).
